# Coaxial Wet Spinning of Boron Nitride Nanosheet-Based Composite Fibers with Enhanced Thermal Conductivity and Mechanical Strength

**DOI:** 10.1007/s40820-023-01236-w

**Published:** 2023-11-20

**Authors:** Wenjiang Lu, Qixuan Deng, Minsu Liu, Baofu Ding, Zhiyuan Xiong, Ling Qiu

**Affiliations:** 1 Tsinghua Shenzhen International Graduate School (TSIGS), Tsinghua University, Shenzhen, 518055 People’s Republic of China; 2 Monash Suzhou Research Institute (MSRI), Monash University, Suzhou, 215000 People’s Republic of China; 3 Faculty of Materials Science and Engineering/Institute of Technology for Carbon Neutrality, Shenzhen Institute of Advanced Technology, Chinese Academy of Sciences, Shenzhen, 518055 People’s Republic of China; 4 Shenzhen Key Laboratory of Energy Materials for Carbon Neutrality, Shenzhen, 518055 People’s Republic of China; 5 School of Light Industry and Engineering, South China University of Technology, Guangzhou, 510614 People’s Republic of China

**Keywords:** Boron nitride nanosheets, Coaxial fiber, Interfacial compression, Nanosheet aligning, Wearable thermal management

## Abstract

Hexagonal boron nitride nanosheets (BNNSs) exhibit remarkable thermal and dielectric properties. However, their self-assembly and alignment in macroscopic forms remain challenging due to the chemical inertness of boron nitride, thereby limiting their performance in applications such as thermal management. In this study, we present a coaxial wet spinning approach for the fabrication of BNNSs/polymer composite fibers with high nanosheet orientation. The composite fibers were prepared using a superacid-based solvent system and showed a layered structure comprising an aramid core and an aramid/BNNSs sheath. Notably, the coaxial fibers exhibited significantly higher BNNSs alignment compared to uniaxial aramid/BNNSs fibers, primarily due to the additional compressive forces exerted at the core-sheath interface during the hot drawing process. With a BNNSs loading of 60 wt%, the resulting coaxial fibers showed exceptional properties, including an ultrahigh Herman orientation parameter of 0.81, thermal conductivity of 17.2 W m^−1^ K^−1^, and tensile strength of 192.5 MPa. These results surpassed those of uniaxial fibers and previously reported BNNSs composite fibers, making them highly suitable for applications such as wearable thermal management textiles. Our findings present a promising strategy for fabricating high-performance composite fibers based on BNNSs.

**Supplementary Information:**

The online version contains supplementary material available at 10.1007/s40820-023-01236-w.

## Highlights

- A core-sheath structured coaxial composite fiber with highly aligned and densely stacked boron nitride nanosheets arrangements in the sheath was successfully fabricated.
- The coaxial fibers have an ultrahigh axial Herman orientation parameter of 0.81, thermal conductivity of 17.2 W m^−1^ K^−1^, and tensile strength of 192.5 MPa.
- The coaxial fibers exhibit intensively potential applications in the wearable thermal management textile.

**Supplementary Information:**

The online version contains supplementary material available at 10.1007/s40820-023-01236-w.

## Graphical abstract

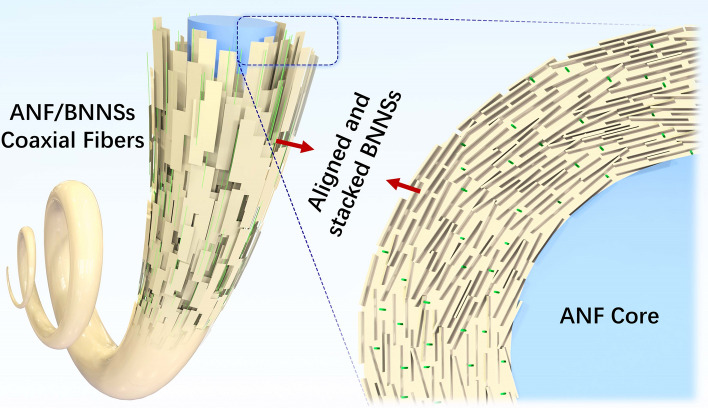

## Introduction

Two-dimensional (2D) nanomaterials, including graphene, black phosphorus, and transition metal oxides or dichalcogenides, have garnered significant attention due to their appealing mechanical, electronic, thermal, and chemical properties [1–4]. The assembly of these 2D nanomaterials into technologically important forms such as films or fibers holds great promise for leveraging their properties in real-world applications [5–7]. One effective approach is wet spinning, which involves injecting highly concentrated colloidal dispersions of 2D nanosheets into a coagulation bath to create macroscopic fiber structures [8–11]. Notably, due to their large aspect ratio of 10^3^–10^4^, 2D nanosheets readily align themselves along the shear flow during uniaxial spinning, resulting in highly oriented microstructures in the axial direction of the fibers [5, 8, 12, 13]. Recently, coaxial spinning has emerged as a method to fabricate 2D nanomaterials-based fibers with core–shell or multilayered structures [14–18]. Compared to uniaxial spinning, coaxial spinning demonstrates distinct advantages in achieving higher nanosheet orientation, which can be facilitated either by the geometry confinement of the shell layer to the core layer [14, 16, 19] or the shearing forces at the core/shell interfaces [20]. Such orientation enables the exploitation of the anisotropy of 2D nanomaterials to enhance the mechanical, electronic, and thermal properties of their fibers, leading to exciting applications such as actuators, thermal management, and wearable devices [21–24].

Research has shown that 2D materials such as graphene oxide and transition metal carbides, nitrides, or carbonitrides (MXenes) can be readily assembled into fibers by wet spinning method. One key reason is that they can be exfoliated into monolayer nanosheets with exceptional in-plane flexibility and hydrophilic functional groups, thereby enabling their effective solution processability [25–27]. 2D hexagonal boron nitride has been extensively explored as an emerging family of 2D materials with remarkable in-plane thermal conductivity (~ 700 W m^−1^ K^−1^) [28, 29] and dielectric properties for thermal management of communication devices (i.e., antenna and radar) without interfering their signal transmission [28, 30–33]. However, the exfoliation process of bulk boron nitride often yields relatively thick yet low functionalized boron nitride nanosheets (BNNSs) due to their strong interlaminar interaction and chemical inertness [29, 34–36], resulting in the much poorer self-assembly capability of pure fibers [34, 37, 38]. To address this, researchers have recently attempted to produce composite fibers of BNNSs/polymer through wet-spinning technique, while the introduction of thermally resistant polymers could compromise the thermal conductivity of BNNSs [39–42]. Moreover, the inherent rigidity of thick BNNSs poses difficulties in achieving highly aligned and densely stacked nanosheet structures within the fibers. Although certain technique such as hot drawing has been employed to enhance BNNSs alignment in the composite fibers, the Herman orientation parameters of BNNSs are still lower than 0.5 [39, 40, 43]. Consequently, the BNNSs/polymer fibers often show a thermal conductivity below 6 W m^−1^ K^−1^ and a mechanical strength less than 150 MPa [39, 42, 44–47]. Therefore, there is an urgent need to further improve the orientation of BNNSs in the fibers to optimize their thermal and mechanical properties for practical applications.

We have recently developed a superacid-based solution system that enables the simultaneous exfoliation and dispersion of BNNSs at ultrahigh concentrations up to 200 mg mL^−1^ (see the attached manuscript that is under peer review). The resulting dispersions of BNNSs are highly compatible with a wide range of wet processing techniques including wet spinning. In this study, we focused on exploring the coaxial wet spinning process for producing BNNSs-based polymer composite fibers. Notably, we discovered a novel mechanism for aligning BNNSs, wherein the compressive forces at the interface between the core and sheath during the hot drawing process effectively induced the alignment and dense stacking of BNNSs in the axial direction, resulting in an ultrahigh Herman orientation parameter of 0.81. Consequently, the thermal conductivity of the coaxial BNNSs/polymer composite fibers reached an impressive value of 17.2 W m^−1^ K^−1^, which is more than twice that (6.6 W m^−1^ K^−1^) of the uniaxial fibers. Additionally, the coaxial fibers exhibited a high tensile strength of 192.5 MPa, facilitating the fabrics weave from BNNSs-based coaxial fibers, which show great potential applications in wearable thermal management textile.

## Experimental Section

### Chemicals and Materials

The hexagonal boron nitride (h-BN) bulk powders were purchased from Qingdao Jinrilai Graphite Co. Ltd. Aramid chopped microfiber yarns (Kevlar 1414) were obtained from DuPont, China. The trifluoromethanesulfonic acid (TfOH) and sodium cholate (SC) were provided by Aladdin Chemical Reagent Plant, China.

### Preparation of Aramid Nanofibers (ANFs)-TfOH Spinning Solutions

0.6 g of dried Kevlar microfiber yarns was added to a small glass bottle containing 15 mL TfOH. Owing to the intrinsic solubility of Kevlar inside strong acids, the ANF/TfOH colloid was prepared in 20 min, stirred by a commercial emulsifier as spun ANF has an average diameter ~ 16.13 ± 3.21 nm (Fig. S1).

### Preparation of ANF/BNNSs-TfOH Spinning Solutions

The preparation of ANF/BNNSs-TfOH dispersions involved the exfoliation of h-BN. As shown in Fig. S2, 0.3 g of dried h-BN powders and 0.05 g of SC were added to a small glass bottle containing 20 mL TfOH. The mixture was continuously high-speed shear-mixed with a 20 min-on and 20 min-off procedure for 4 h. This method successfully fabricated exfoliated BNNSs with an average lateral size of ~ 1.0 um (Fig. S3). Subsequently, 0.3 g of dried Kevlar microfiber yarns was added and continuously mixed for 20 min, resulting in the formation of 50 wt% ANF/BNNSs viscous spinning solutions. The obtained solutions were left standing for 24 h to remove the mixed bubbles before spinning. Using a similar method, highly concentrated ANF/BNNSs spinning dopes (10, 20, 30, 40, 50, 60, 70, 80, and 90 wt%) could be prepared with a fixed ANF content of 0.3 g and the corresponding BNNSs loadings.

### Preparation of ANF/BNNSs Coaxial Fibers

**Fig. 1:**
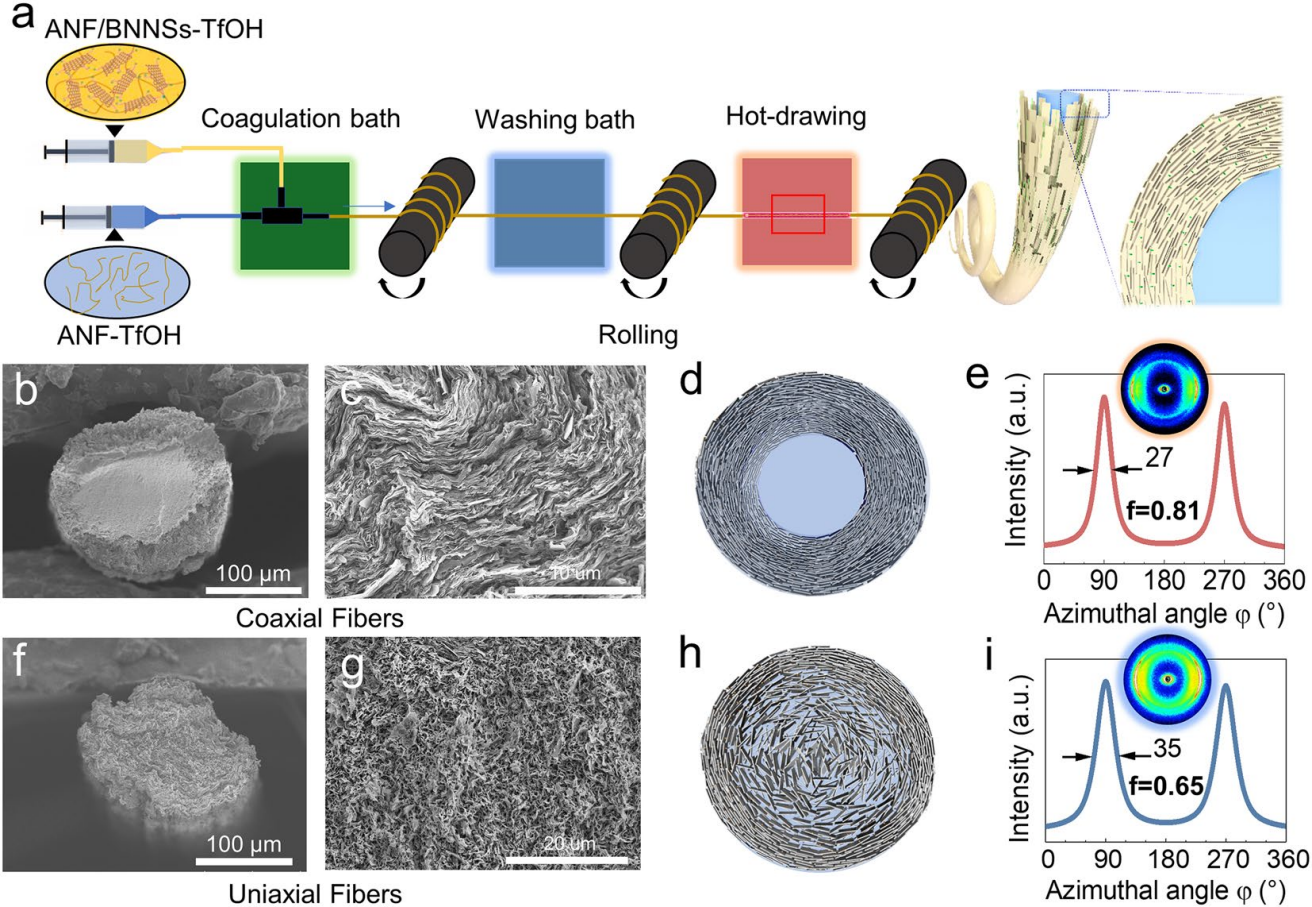
**a** Schematic illustrating the fabrication of core-sheath fiber by a coaxial wet-spinning technique. SEM images showing the cross-sectional morphology of **b**,** c** the coaxial fibers and **f**, **g** the uniaxial fibers prepared with a tensile strain of 12%. The schematics showing **d** the highly axial oriented arrangement of BNNSs spanning the entire sheath of coaxial fibers and **h** the shallow surface-depth BNNSs axial oriented arrangement of uniaxial fibers. 2D WAXS images with curves of the scattering intensity as a function of azimuthal angle (φ) for **e** coaxial and **i** uniaxial BNNSs fibers

The coaxial fibers were prepared using a setup consisting of a coaxial needle mounted with a polytetrafluoroethylene tubular extension and fed with two syringe pumps (Fig. 1a). The coaxial needle consisted of an internal needle (22G, channel diameter ~ 420 μm) and an external needle (17G, channel diameter ~ 1110 μm). For coaxial fibers with different BNNSs contents, specific concentrated viscous ANF/BNNSs solutions were connected to the sheath channel, while pure viscous ANF solutions were linked with the core entrance. The extrusion flow speeds were set to 0.2 mL min^−1^ (core flow) and 0.4 mL min^−1^ (sheath flow). During the wet-spinning process, the coaxial needle was submerged into a water-based coagulation bath. The extruded wet filaments were dragged and collected using a roll (rotation speed 10 rpm, roll diameter 10 cm). After a water-based washing bath to remove the residual TfOH, gel-like coaxial filaments were obtained. Finally, the coaxial fibers were successfully prepared by conducting the hot drawing process, which involved stretching and drying the filaments through a drying oven at 80 °C for 15 min with a specific tensile strain (0, 4%, 8%, 12%, 16%).

### Preparation of ANF/BNNSs Uniaxial and Hollow Fibers

To prepare ANF/BNNSs uniaxial fibers, different concentrated ANF/BNNSs solutions were directly wet-spun through a uniaxial needle (17G, channel diameter ~ 1110 μm). For the preparation of hollow ANF/BNNSs fibers, the coaxial needle (22G/17G) was used, extracting only the ANF/BNNSs suspensions through the sheath channel. Both of the uniaxial and hollow fibers were underwent the same washing and hot-drawing procedures as the preparation of the coaxial fibers.

### Characterizations

The microstructural morphologies of the fabricated fibers were examined using field-emission scanning electron microscopy (SEM, SU8010, Hitachi, Japan) at an accelerating voltage of 5 kV. To analyze the cross-sectional morphologies of the fibers, cryo-fracturing using liquid nitrogen was performed. The exact BNNSs loadings of coaxial fibers were determined through thermogravimetric analysis (TGA, TG209F3, NETZSCH, Germany) in a nitrogen atmosphere within a temperature range of 100–900 °C at a heating rate of 10 °C min^−1^. The specific heat volumes of the fibers were examined by differential scanning calorimetry (DSCQ2000, TA Instruments, USA) under a nitrogen atmosphere with the temperature increased from 20 to 250 °C at a heating rate of 10 °C min^−1^. The crystal structures of the h-BN and BNNSs powders were investigated using an automated X-ray powder diffractometer (XRD, SmartLab, Japan), at a scan rate of 20° min^−1^ with a 2*θ* range of 10°–80°. Fourier transform infrared (FTIR) spectroscopy was recorded on a Nicolet 8700 spectrometer in the range of 4000–400 cm^−1^. X-ray photoelectron spectroscopy (XPS) analysis was conducted using an Escalab 250xi (Al Kα, *hυ* = 1486.6 eV). The Herman orientation parameters of BNNSs within the fibers are determined by 2D wide‑angle X‑ray scattering (WAXS) measurements using an Anton Paar SAXS point 2.0 system (Anton Paar, Graz, Austria) equipped with a microsource X-ray source (Cu Kα radiation, wavelength 0.15418 nm). The mechanical properties of the fibers were tested by performing uniaxial tensile tests at room temperature using an Instron 5566 universal testing machine (Norwood, MA, USA) equipped with a 50 N load cell. For the evaluation of mechanical properties, the fibers were assumed to be cylindrical, and their diameters were evaluated by SEM images using ImageJ software. At least 5 specimens were tested for each configurated fibers. The resilience of the fibers was also recorded under a five-cyclic test. The thermal conductivities of the fibers were characterized by the transient electro-thermal (TET) technique [48, 49]. The surface temperature of textiles woven by the coaxial fibers and the pure ANF fibers in the application display was recorded using an Fotric 255 s infrared thermograph.

## Results and Discussion

### Preparation and Characterization of Coaxial and Uniaxial BNNSs-based Composite Fibers

Figure 1a depicts the schematic of the fabrication of BNNSs/polymer fibers using a coaxial wet spinning process. ANF was chosen as the polymer matrix due to its exceptional dispersion capability in TfOH superacids. The coaxial fibers were created by utilizing two different dopes: pure ANF dispersions in TfOH as the core and mixed dispersions of ANF and BNNSs in TfOH as the sheath. Unless otherwise specified, the BNNSs content in the mixed dispersions was 50 wt%. For the fabrication of coaxial fibers, the spinning dopes were injected into a water-based coagulation bath and subsequently washed to eliminate any remaining TfOH residues. The resulting gel fibers were then dried under hot drawing, which has been widely used for promoting the axial alignment of 2D nanosheets within fibers.

Figure 1b displays the cross-sectional morphology of the coaxial ANF/BNNSs fibers dried at a 12% tensile strain, clearly demonstrating the presence of a distinct core-sheath structure. A closer examination (Fig. 1c) reveals a pronounced axial-oriented and densely stacked arrangement of BNNSs spanning the entire 40-µm-wide sheath. The BNNSs exhibit a circular arrangement surrounding the round ANF core, with the nanosheet edges perpendicular to the cross-section of the fibers. This configuration resembles the wrapping of annual rings around a tree trunk, as depicted in Fig. 1d. The orientation of BNNSs was quantitatively characterized by wide-angle X-ray scattering (WAXS) [25, 50]. The characteristic arcs in the 2D scattering patterns of the coaxial fibers indicate the high orientation of BNNSs along the fiber axis (Fig. 1e). The calculated Herman orientation parameter can be as high as 0.81, surpassing other reported BNNS-based composite fibers (Fig. S4) [39, 40, 43].

To investigate the impact of coaxial structure, we fabricated the uniaxial ANF/BNNSs fibers for comparison. As shown in the cross-sectional morphology of the uniaxial fibers, BNNSs also display axially oriented and densely stacked configuration similar to that of coaxial fibers within the surface 15-µm-depth area (Fig. 1g). However, BNNSs tend to exhibit random when approaching the center of the fibers, which is illustrated in Fig. 1h. Correspondingly, the Herman orientation parameter of the uniaxial fibers reach only 0.65 (Fig. 1i), considerably smaller than that of the coaxial fibers. Given that both the coaxial and uniaxial fibers were prepared using spinning needles of the same diameter, we can preliminarily conclude that the presence of the ANF core is essential in facilitating the axial alignment and dense stacking of BNNSs in the coaxial fibers. This is further supported by analyzing the structure of hollow ANF/BNNSs fibers fabricated through coaxial spinning with the absence of ANF core. It is evident that BNNSs are disordered within the hollow fibers, and the Herman orientation parameter of BNNSs is merely 0.16, further confirming the key role of ANF core in promoting the nanosheet orientation (Fig. S5).

### Evolution Process of BNNSs Alignment during Hot Drawing

**Fig. 2:**
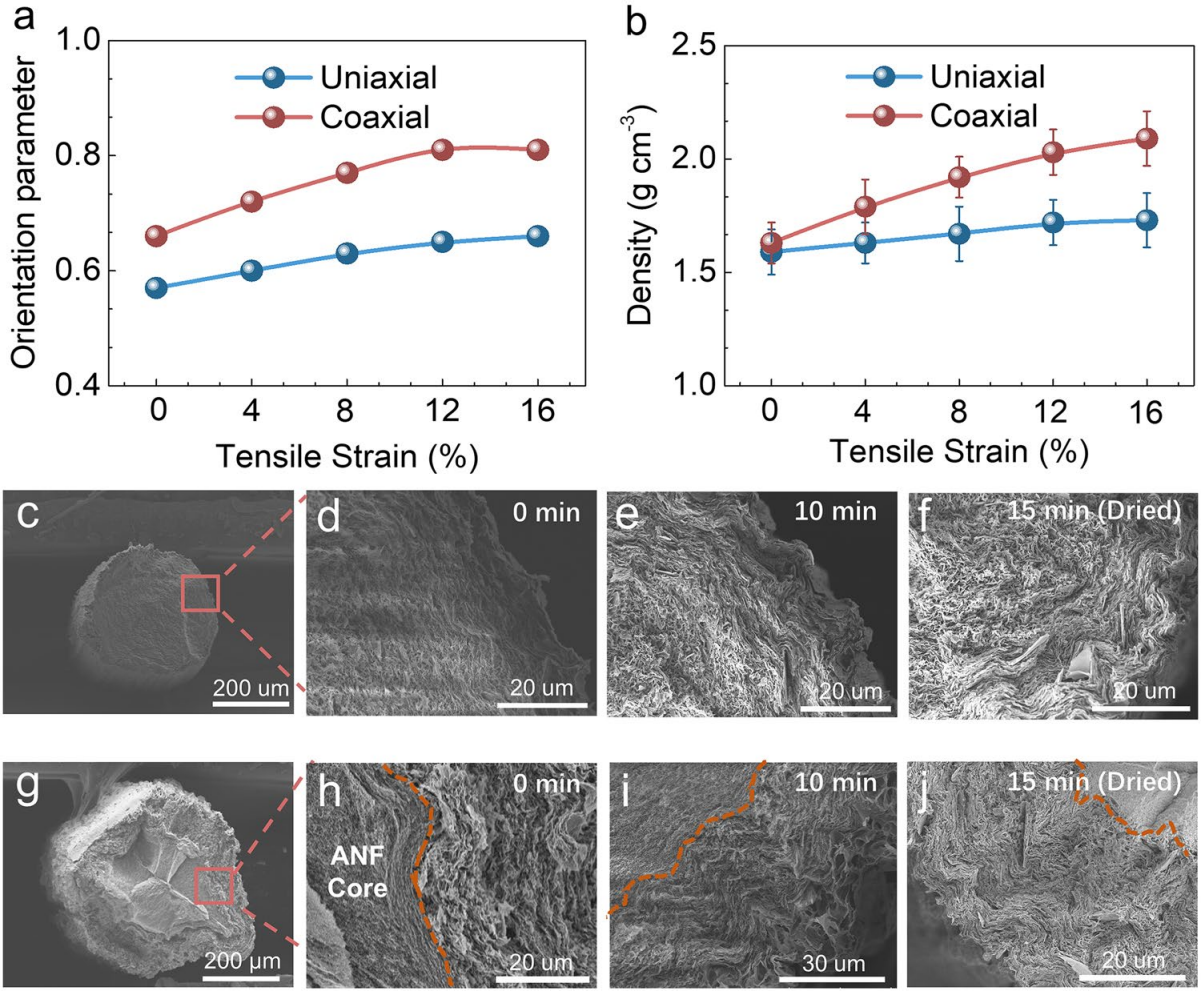
**a** The Herman orientation parameters and **b** densities as a function of tensile strain for the coaxial and uniaxial BNNSs fibers. SEM images showing the cross-sectional morphologies of freeze-dried tensile **c** uniaxial and **g** coaxial fibers under the corresponding different dried time of **d**, **h** 0 min, **e**, **i** 10 min, **f**, **j** 15 min

To understand the orientation process of BNNSs, we first studied the influence of the tensile strains in the hot drawing on the orientation of BNNSs in the fibers. It is noted that a considerable orientation degree of BNNSs can be achieved for coaxial fibers (0.66) and for uniaxial fibers (0.57) without hot drawing due to the flow-induced aligning of BNNSs in the wet spinning [19, 20]. As shown in Fig. 2a, b, the Herman orientation parameters and densities of coaxial fibers would further increase with the increase of tensile strains and can reach as high as 0.81 and 2.09 g cm^−3^ at the strain of 12%, respectively. These are 23% and 28% higher than those of the fibers without tension. In comparison, although the orientation parameters and densities of uniaxial fibers can also increase with the increase of tensile strains, the enhancements are only 14 and 8%, respectively, under 12% tensile strain, and the values are 0.65 and 1.72 g cm^−3^. This suggests that the enhancement of BNNSs orientation is more pronounced in coaxial fibers than in uniaxial fibers during the hot drawing process. The cross-sectional SEM images of the fibers further confirmed the enhanced orientation and dense stacking of BNNSs in coaxial fibers after hot drawing (Figs. S6, S7) (12% strain was selected as optimal processing parameter owing to the maximum tensile strength of the fiber, detail see Fig. S8).

We further investigated the structural evolution process of the coaxial and uniaxial fibers under a 12% tensile strain during hot drawing. Freeze-dried SEM images of the coaxial and uniaxial fibers after different drying time (0–15 min) are shown in Fig. 2c–j. For the uniaxial fibers, the orientation and densification of BNNSs appear to initiate from the fiber surface, with the thickness of the oriented BNNSs layer gradually increasing from nearly 0 um at 0 min to approximately 10 μm at 10 min and approximately 15 μm at 15 min (Figs. 2c–f, S9). Nevertheless, at the final drying stage (15 min), a significant portion of disordered BNNSs remains in the interior of the uniaxial fibers. In contrast, the coaxial fiber exhibits a distinct process for the orientation and densification of BNNSs, starting from the core/sheath interface and progressing toward the surface. As depicted in Figs. 2g–j and S10, BNNSs were disordered near the interfaces at the beginning of the drying. After 10 min drying, BNNSs became oriented near the interface but remained disordered at the surface. Eventually, BNNSs were oriented throughout the entire cross-section of the fibers. These results indicate that the orientation of BNNSs during the drying process begins from the surface for the uniaxial fibers (outside-in process), while it commences from the core/sheath interface for the coaxial fibers (inside-out process). This indicates the potential influence of the interface of the coaxial fiber on the BNNSs orientation. Further, we used different sizes of in and out needles to exam the influence of the thickness on the orientation parameter (Fig. S11). It shows no differences in the orientation parameter, emphasizing the presence of core-sheath interface can induce the well-aligned structure in various scenarios.

### Finite Element Simulation of the BNNSs Aligning Mechanism in Coaxial Fibers

**Fig. 3:**
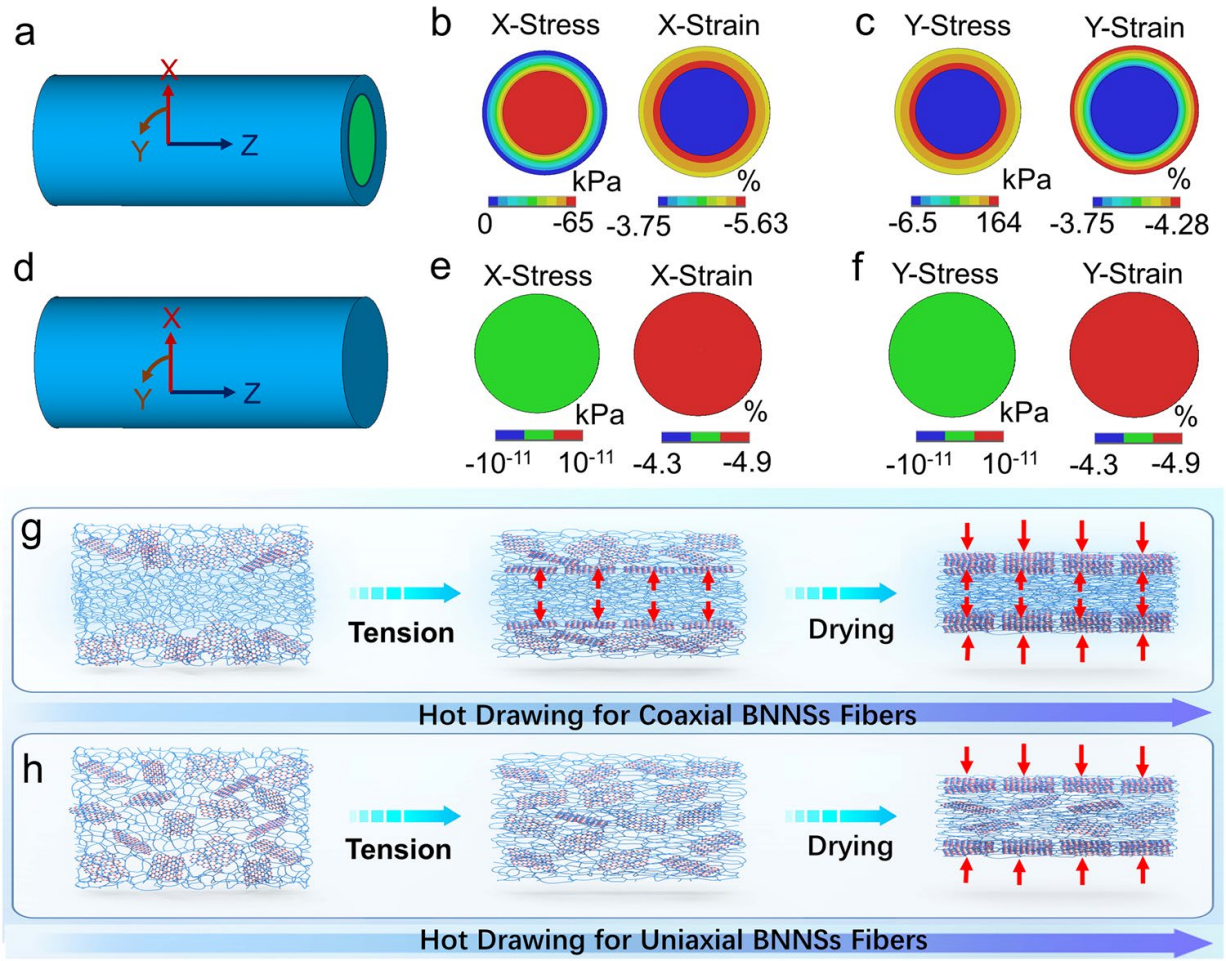
Two types of cylinder modes of **a** coaxial and **d** uniaxial fibers for FEM simulation. The FEM simulation results indicating the cross-sectional X/Y-axial stresses and strains distribution color contours of **b**,** c** coaxial fibers and **e**,** f** uniaxial fibers with a 12% tensile strain. Schematics illustrating the evolutions of BNNSs axial aligning and dense stacking for **g** coaxial fibers and **h** uniaxial fibers during the hot drawing procedure. The red arrows indicate the interfacial compressive stress of coaxial fibers when tension and the radial capillary contraction of both fibers when drying

To investigate the orientation mechanism of BNNSs, finite element model (FEM) simulations were conducted to analyze the mechanical conditions of the spun fibers (see details in Supporting Information). In line with the experimental setup, two types of cylinders, namely uniaxial and coaxial (Fig. 3a, d), were created and subjected to a 12% strain load (see details in supporting information). The material properties utilized in the FEM were determined through experimental measurements (Fig. S12; Table S1), including dimensions, Young's modulus, and Poisson's ratio.

Considering the dynamic changes in material properties during the hot drawing process, our analysis primarily focuses on the mechanical conditions of the wet filaments at the initiation of hot drawing. Figure 3b, c illustrates the distributions of stresses and strains at the cross section of the fiber in radial direction (*X*) and circumferential direction (*Y*). In *X* direction, the surface of the coaxial cylinder experiences minimal stress and strain, which gradually increase toward the core and peak at the core/sheath interface (Fig. 3b). This behavior can be attributed to the sheath's larger Poisson's ratio, leading to greater radial shrinkage compared to the core and resulting in compressive stress. In the *Y* direction, the stress is maximum at the core/sheath interface and the strain is maximum at the surface of the coaxial cylinder (Fig. 3c). Notably, the difference in strain between the core and the sheath at the interface is more pronounced in the *X* direction than in the *Y* direction (− 3.75% to − 5.63% vs − 3.75% to − 3.99%). The underlying reason is that the core with a smaller Poisson's ratio can counteract the circumferential contraction of the sheath. In contrast, as shown in Fig. 3e, f, the stresses and strains in the *X* and *Y* directions of the uniaxial cylinder are uniformly distributed across the entire cross section of the fibers.

Based on the simulation results, we propose the following process for the orientation of BNNSs in the fibers. As illustrated in Fig. 3g, in the case of wet coaxial filaments, the hot drawing process induces compressive stress at the interfaces, which is exerted on the sheath throughout the entire drying process due to the differing Poisson's ratios of the core and sheath. Additionally, a capillary compressive stress naturally develops on the surface of the sheath during drying. These two compressive stresses gradually align and orient the BNNSs along the axial direction of the coaxial fiber [51–53]. In contrast, as illustrated in Fig. 3h, the uniaxial fibers only experience capillary compressive stress. Consequently, the degree of BNNSs orientation is higher in the coaxial fibers. The role of interfacial compressive stress is further supported by the higher BNNSs orientation observed in coaxial fibers subjected to larger tensile strains during hot drawing, which can induce larger interfacial compression to promote the orientation (Fig. S13).

### Enhanced Thermal Conductive and Mechanical Properties of Coaxial Fiber

**Fig. 4:**
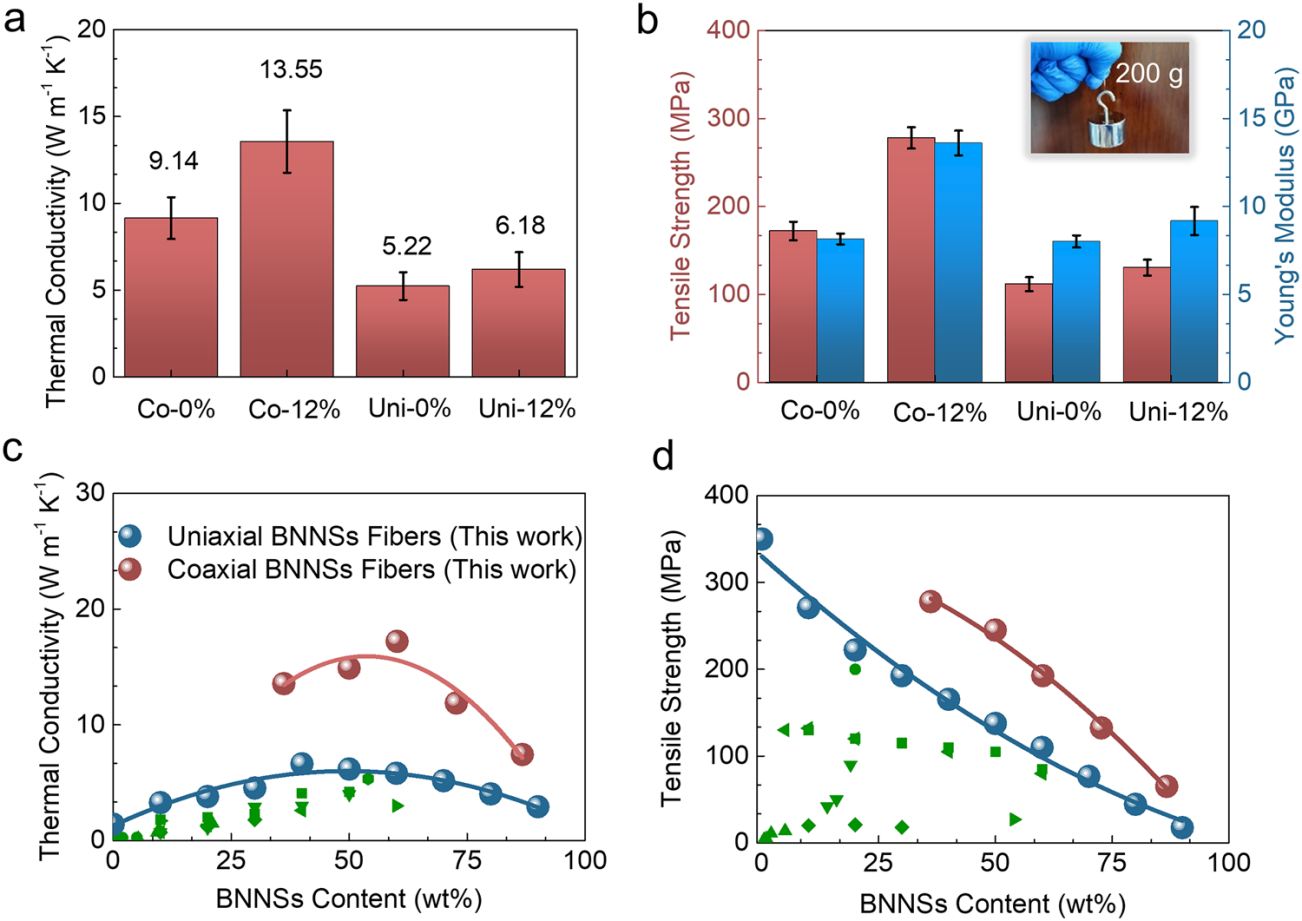
**a** Compared thermal conductivities and **b** mechanical properties of the coaxial and uniaxial fibers with BNNSs content of 50 wt%. Samples Co-0%/Uni-0% and Co-12%/Uni-12% stand for the coaxial/uniaxial fibers with the corresponding 0% and 12% tensile strains while hot drawing. The inserted photograph shows the Co-12% coaxial fiber hanging a 200 g weight. Comparisons of our data with results reported elsewhere as **c** thermal conductivity and **d** tensile strength. The green data are the results reported in other references [38–42, 44–47, 61], which are listed in Table S2

The high orientation of BNNSs is found to significantly enhance the thermal conductivity of coaxial fibers. As depicted in Fig. 4a, the coaxial fibers prepared under a 12% tensile strain (Co-12%) in hot drawing exhibit a substantial improvement in thermal conductivity, with an increase of 48% from 9.14 to 13.5 W m^−1^ K^−1^. On the other hand, the uniaxial fibers prepared under the same tensile strain (denoted by Uni-12%) only experience a modest 18% rise in thermal conductivity, reaching 6.18 W m^−1^ K^−1^ from an initial value of 5.22 W m^−1^ K^−1^. The thermal conductivity ranking of these fibers shows a strong correlation with the orientation degree of BNNSs, highlighting the significant impact of BNNSs orientation on the thermal conductivity of fibers. The Co-12% fiber exhibits 4.41 W m^−1^ k^−1^ improvement in thermal conductivity than the Co-0 sample. This can be attributed to the highly oriented and densely stacked BNNSs that form a connected network throughout the entire sheath of coaxial fibers, establishing a continuous thermal conductive pathway that facilitates efficient heat transfer along the fiber axis (Fig. S14a) [54, 55]. In contrast, achieving such a pathway is challenging in uniaxial fibers due to the orientation of BNNSs mainly occurring near the surface region, and the presence of pores between loosely stacked BNNSs in the central region of the fibers (Fig. S14b).

The mechanical properties of the coaxial fibers exhibited more significant improvements compared to the uniaxial fibers. When subjected to a 12% tensile strain during the hot drawing process, the tensile strength and Young's modulus of the coaxial fibers increased substantially, rising from 172.1 to 277.9 MPa and 8.2 to 13.6 GPa, respectively. On the other hand, the tensile strength and Young's modulus of the uniaxial fibers only experienced slight increases, going from 111.3 to 137.5 MPa and 8.0 to 9.2 GPa, respectively. Notably, the optimal tensile strength (277.9 MPa) and Young's modulus (13.6 GPa) achieved by the coaxial fibers were nearly twice as large as those of the uniaxial fibers (137.5 MPa and 9.2 GPa). The enhancement can be attributed to the high orientation and dense stacking of BNNSs [56–58]. Additionally, the ANF (aligned nanofiber) core, with its high tensile strength (365.2 MPa) and Young's modulus (14.3 GPa), plays a crucial role in enhancing the mechanical properties of the coaxial fibers (Fig. S15) [59, 60]. The tensile fractured morphologies of the coaxial fibers, as depicted in Fig. S16, clearly demonstrate the reinforcement provided by the nanofiber.

We further examined the thermal and mechanical properties of coaxial fibers and uniaxial fibers with varying BNNSs loading (Figs. S17–S19) and compared them with those of previous studies. As depicted in Fig. 4c, the thermal conductivity of coaxial fibers increases with the content of BNNSs and reaches its peak at a sheath loading of 70 wt%. However, further increasing the BNNSs loading does not necessarily enhance the thermal conductivity of the fiber due to the decreased orientation of BNNSs at high loading (Fig. S20). Similar relationship between the thermal conductivity and the BNNSs content is also found for uniaxial fibers (Figs. 4c, S21). The optimized thermal conductivity of the coaxial fibers (17.2 W m^−1^ K^−1^ BNNSs content 70 wt% at outer sheath) three times of the maximum thermal conductivity achieved by the uniaxial fibers (6.74 W m^−1^ K^−1^ at overall BNNSs content 70 wt%). Moreover, it represents a record-high value among the reported BNNSs-based fibers (typically below 6 W m^−1^ K^−1^) in other studies [38–42, 45, 47, 61]. Additionally, both the tensile strengths of coaxial and uniaxial fibers gradually decrease with increasing BNNSs loading. However, the tensile strength of the coaxial fibers remains higher than that of the uniaxial fibers (Fig. 4d). Benefiting from the presence of a robust ANF core, the coaxial fibers exhibit a high tensile strength of 192.5 MPa at the optimized thermal conductivity (BNNSs loading of approximately 70 wt% in the sheath, corresponding to 60 wt% in coaxial fibers). This tensile strength is superior than those (below 150 MPa) reported in other studies with different BNNSs contents (5–50 wt%) [39, 42, 44–47].

### Cooling Textile Application

**Fig. 5:**
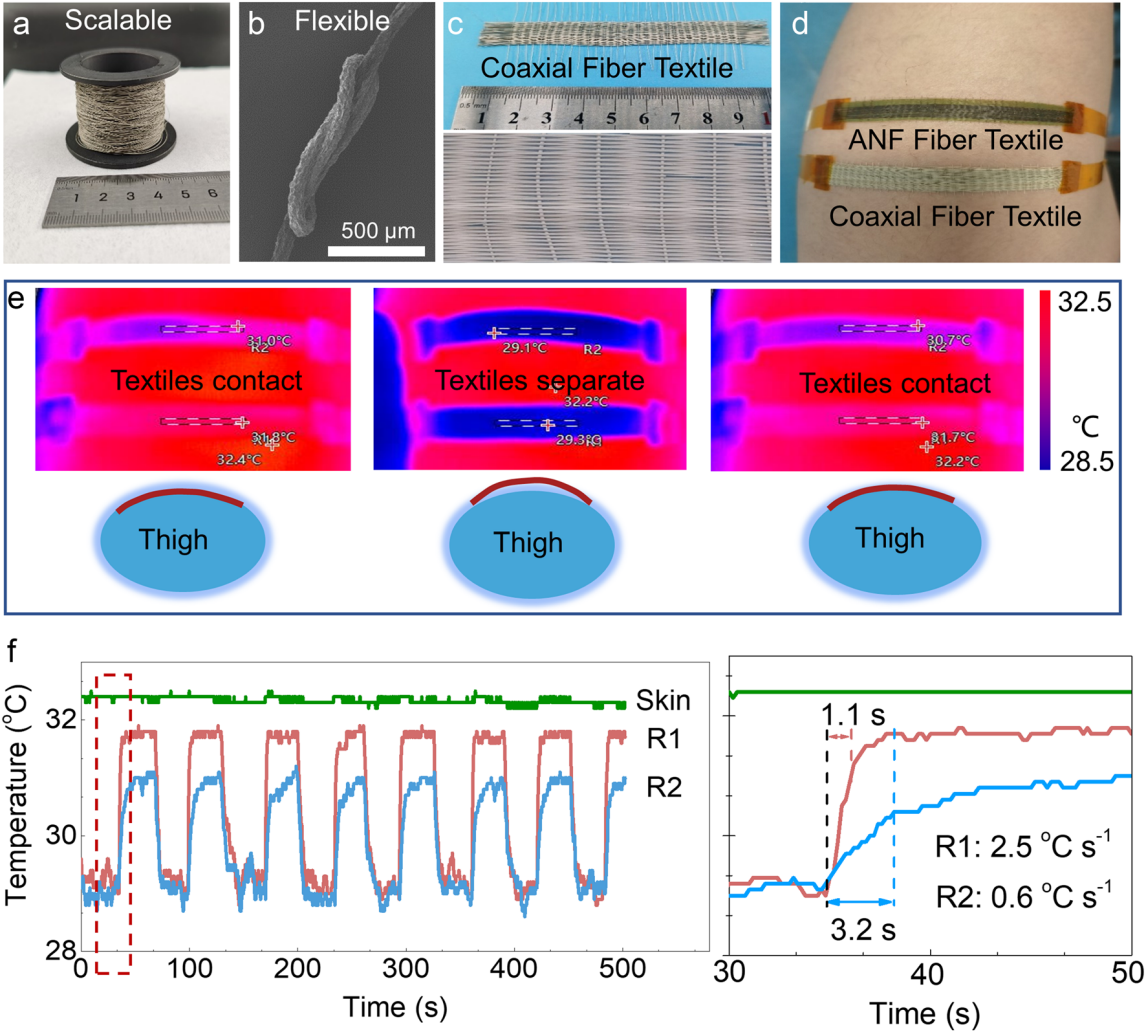
Compared thermal cooling properties of coaxial fiber and ANF fiber fabrics. **a** Photograph image showing the coaxial fibers winding on a yarn bobbin, indicating the scalability of preparation. **b** SEM image showing the knot of coaxial fibers, revealing the flexibility. **c** Photograph image showing the textiles woven by coaxial fibers. **d** Strip-shaped fabrics of ANF and coaxial fibers attached to human skin with both ends firmly sticked to the thigh. **e** Infrared images and corresponding schematics showing the surface temperatures of ANF and coaxial fibers textiles under a cyclic contact-separate-contact process. The R1 and R2 represent the average temperatures detected by infrared camera within the enclosed rectangular areas of ANF and coaxial fiber textiles, respectively. **f** Surface temperatures of the textiles and human skin as a function of time under the continuous cyclic textile contact-separate process and **g** the enlarge graph showing the heating rates for the textiles while contacting the skin

Woven fabrics supply effective protection and appropriate mass exchange between human skin and environment. To use highly thermal conductive fiber for fabrics can promote the thermal transfer for personal cooling. Here, we investigated the potential of coaxial ANF/BNNSs composite fibers for such application. Utilizing the developed coaxial wet-spinning method, we successfully produced continuous coaxial ANF/BNNSs fibers (Fig. 5a). Specifically, we focused on the coaxial fibers with a 70 wt% BNNSs loading sheath, which exhibited the highest thermal conductivity. These fibers possess mechanical flexibility, allowing for easy knotting, and were subsequently woven into wearable fabrics suitable for cooling textiles (Fig. 5b, c).

The cooling performance of the coaxial composite fibers was assessed using infrared camera. Fabrics composed of pure ANF fibers were produced and utilized as a reference. Both fabric strips were meticulously affixed to the human skin, with the ends securely fastened to the thigh. This arrangement enabled cyclic contact and separation of the middle sections of the fabrics from the skin by applying pressing forces (Fig. 5d). The temperature changes on the surface of the textiles during the cyclic process were examined (Fig. 5e). Upon contact with the skin at a temperature of 32.4 °C, the coaxial fiber fabrics experienced a rapid temperature rise from 29.1 to 31.8 °C within 1.1 s. Conversely, once separated from the skin, they quickly returned to their original temperature of ~ 29 °C. This indicates efficient heat exchange between the coaxial fiber fabrics and the skin and between the fabrics and the air environment. Essentially, the coaxial fiber fabrics can function as a medium to facilitate heat transfer from high-temperature skin to a low-temperature environment (Fig. S22). In contrast, when ANF fiber fabrics with poor thermal conductivity were employed, the fabric temperature slowly increased to only 31.0 °C after 3 s of skin contact (Fig. 5f). The temperature rise rate was calculated to be merely 0.6 °C s^−1^, significantly lower than the 2.5 °C s^−1^ observed with coaxial fiber fabrics. The coaxial fiber textile's rapid cooling performance renders it highly suitable for wearable sports fabrics that involve cyclic contact and separation with the skin during activities such as walking or engaging in sports [62–64].

## Conclusions

In summary, we have successfully developed a coaxial wet spinning method to fabricate BNNSs-based composite fibers with exceptional orientation and enhanced properties. By leveraging compressive stresses at the core-sheath interface during the hot drawing process, we achieved an ultrahigh Herman orientation parameter of 0.81, surpassing previous reports. The coaxial fibers exhibited a remarkable thermal conductivity of 17.2 W m^−1^ K^−1^, more than double that of uniaxial fibers. Additionally, the fibers demonstrated a high tensile strength of 192.5 MPa, enabling their use in wearable thermal management textiles. This study highlights the importance of the coaxial structure and the core’s role in promoting BNNSs alignment. Our findings contribute to the development of high-performance BNNSs-based fibers and open up new opportunities for their application in various fields, including thermal management, actuators, and wearable devices.

## Supplementary Information

Below is the link to the electronic supplementary material.

Supplementary file1 (PDF 2243 kb)
