# Supplementary material for: Coaxial Wet Spinning of Boron Nitride Nanosheet-Based Composite Fibers with Enhanced Thermal Conductivity and Mechanical Strength
Source: Nanomicro Lett. 2023 Nov 20;16:25. doi: 10.1007/s40820-023-01236-w (PMC10661126; doi:10.1007/s40820-023-01236-w)
Supplement: Supplementary file 1 — Supplementary file1 (PDF 2243 kb) [file 40820_2023_1236_MOESM1_ESM.pdf]

Supporting Information for

Coaxial Wet Spinning of Boron Nitride Nanosheet-Based Composite Fibers with Enhanced Thermal Conductivity and Mechanical Strength

Wenjiang Lu[1,#], Qixuan Deng[1,#], Minsu Liu[2], Baofu Ding[3, 4], Zhiyuan Xiong[5,*], Ling Qiu[1,*]

[1]Tsinghua Shenzhen International Graduate School (TSIGS), Tsinghua University, Shenzhen 518055, P. R. China

[2]Monash Suzhou Research Institute (MSRI), Monash University, Suzhou 215000, P. R. China

[3]Faculty of Materials Science and Engineering/Institute of Technology for Carbon Neutrality, Shenzhen Institute of Advanced Technology, Chinese Academy of Sciences, Shenzhen 518055, P. R. China

[4]Shenzhen Key Laboratory of Energy Materials for Carbon Neutrality, Shenzhen 518055, P. R. China

[5]School of Light Industry and Engineering, South China University of Technology, Guangzhou 510614, P. R. China

[#]Wenjiang Lu and Qixuan Deng contributed equally to this work.

[*]Corresponding authors. E-mail: ling.qiu@sz.tsinghua.edu.cn (Ling Qiu); xzyscut@scut.edu.cn (Zhiyuan Xiong)

S1 Finite Element Modelling (FEM)

The purpose of the finite element model is to better understand the undermine mechanism of how the ANF core affecting the sheath BNNSs orientation within the coaxial designed fibers. The finite element modelling has been carried out with the ANSYS 19 software. The 3D FE model of the coaxial fiber is built in a cylindrical coordinate system and combined with two parts including the inner core (MESH200) and the outer sheath (SOLID185). The two parts are completely bonded by shared nodes as the core-sheath interface of the coaxial fibers are seamlessly connected by the ANF network. For simulating the deformation under tensile load, one end of the cylindrical model is fixed, and the other end is applied to a tensile strain of 12%. The validation of the FE model is done by modifying the material property of different components to check whether the FE model under tension has the conjectured interfacial stress and strain that can explain the effect of BNNSs axial orientation and stacking. For comparison, the 3D FE model of the uniaxial fiber is also built as a single cylinder with

the same material properties of the sheath component of coaxial fiber. The material properties of all components used for FE models are experimentally determined and provided in Table S1. The Young's Modulus is derived from the stress-strain curves of wet filaments (Fig. S9). The Poisson's ratios of fibers are calculated by measuring the diameter change of the wet filaments after a certain stretch strain using a light microscope. The sheath BNNSs are uniformly but randomly distributed in a sparser ANF network compared with inner core (Fig. S5a). This will lead to the ANF core with a higher Young's modulus and the sheath with a larger Poisson's ratio for the wet coaxial fibers. Simulations are performed to obtain the distributions of Von Mises stress and strain under different axials inside the fibers.

S2 Supplementary Figures and Tables

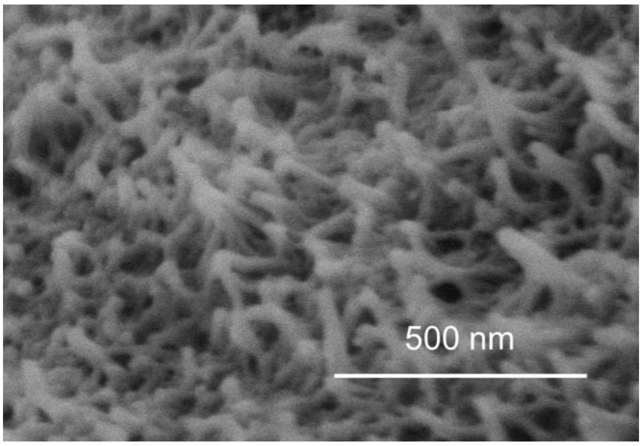

Fig. S1 SEM image of ANF for measuring the average diameter of the nanofibers

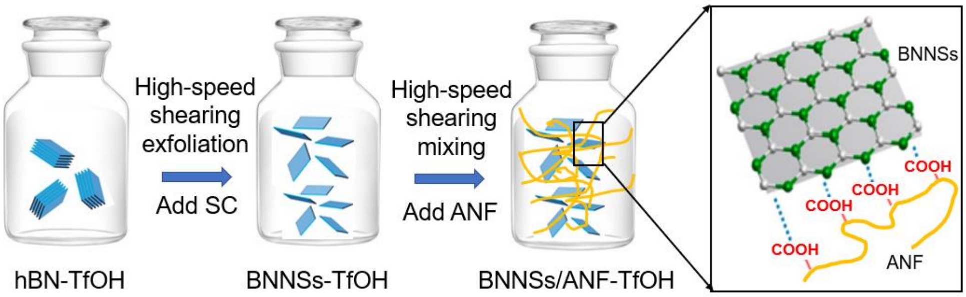

Fig. S2 Schematic of the exfoliation of BNNSs and the preparation of BNNSs/ANF-TfOH dispersions

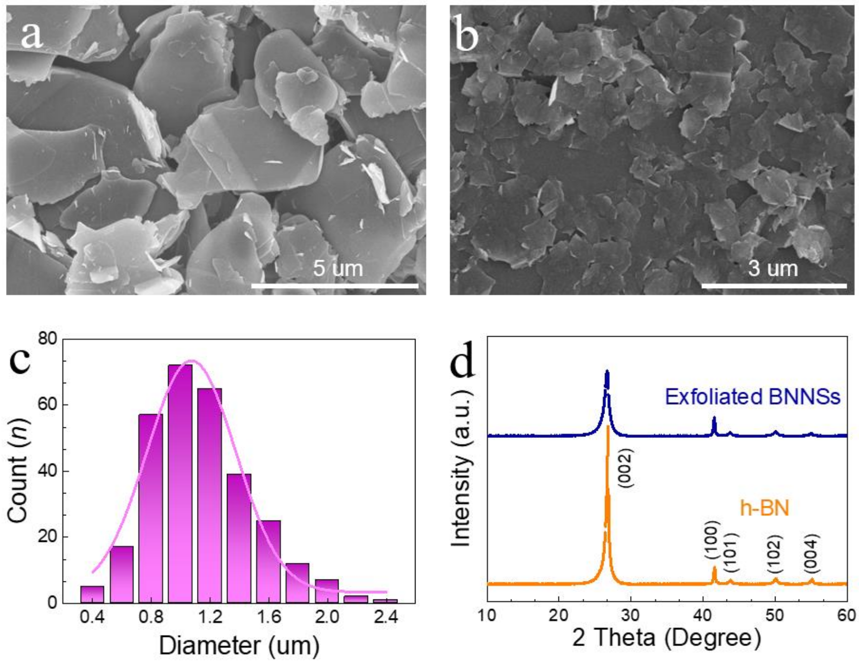

Fig. S3 (**a**) SEM image of h-BN powders with a lateral size of 5-15 μm and thicknesses of several hundred nanometers. (**b**) SEM image showing the much smaller and thinner BNNSs nanoplates with **c** an average lateral size of ~1.0 um. (**d**) XRD patterns of h-BN and BNNSs. The much broader characteristic peak of BNNSs is attributed to the decrease of the thickness-to-lateral size ratio, revealing the h-BN powders were successfully exfoliated to thin BNNSs nanosheets

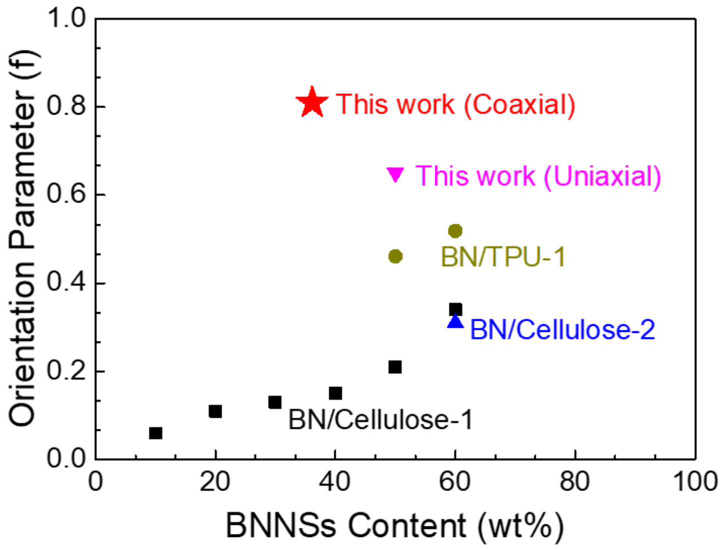

Fig. S4 Comparison of our Herman orientation parameters with those reported in other studies on BNNS-based fibers [S1-S3]

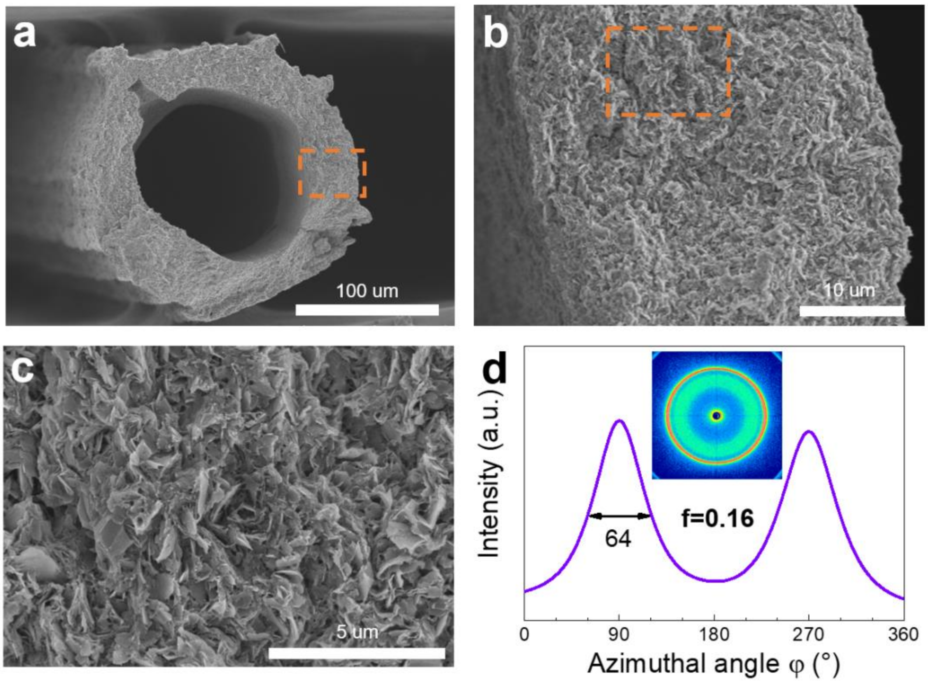

Fig. S5 (**a-c**) SEM images showing the cross-sectional morphologies of ANF/BNNSs hollow fibers, and (**d**) the 2D WAXS image with curve of the scattering intensity as a function of azimuthal angle (φ)

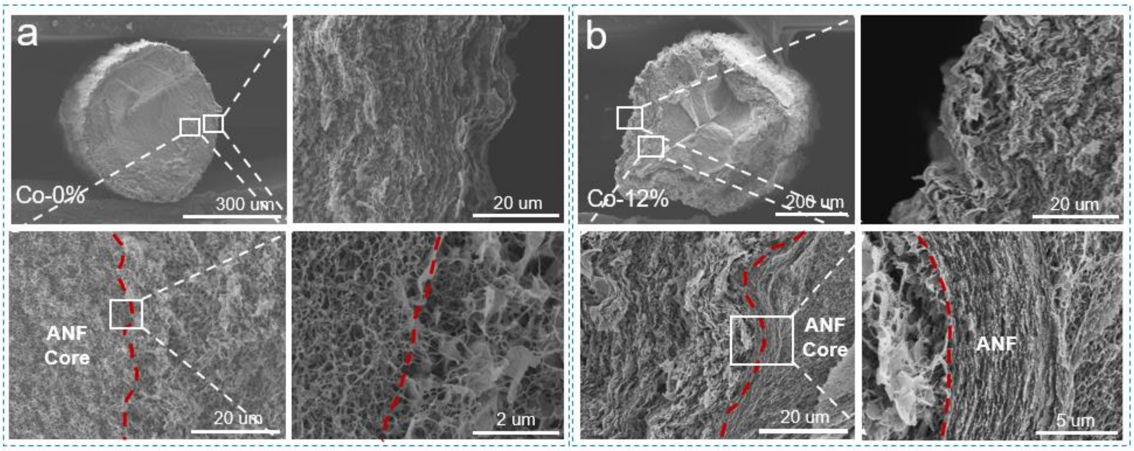

Fig. S6 SEM images showing the cross-sectional morphologies of freeze-dried ANF/BNNSs coaxial wet filaments (**a**) without and (**b**) with a 12% tensile strain. The coaxial fibers without tension show that the core is a pure ANF network and the sheath is a sparser ANF network embedded with randomly distributed BNNSs. When the coaxial fiber is applied with a 12% tensile strain, the BNNSs axial orientation arrangement preferentially happens at the interface adjacent area and meanwhile a dense ANF thin layer also shows up

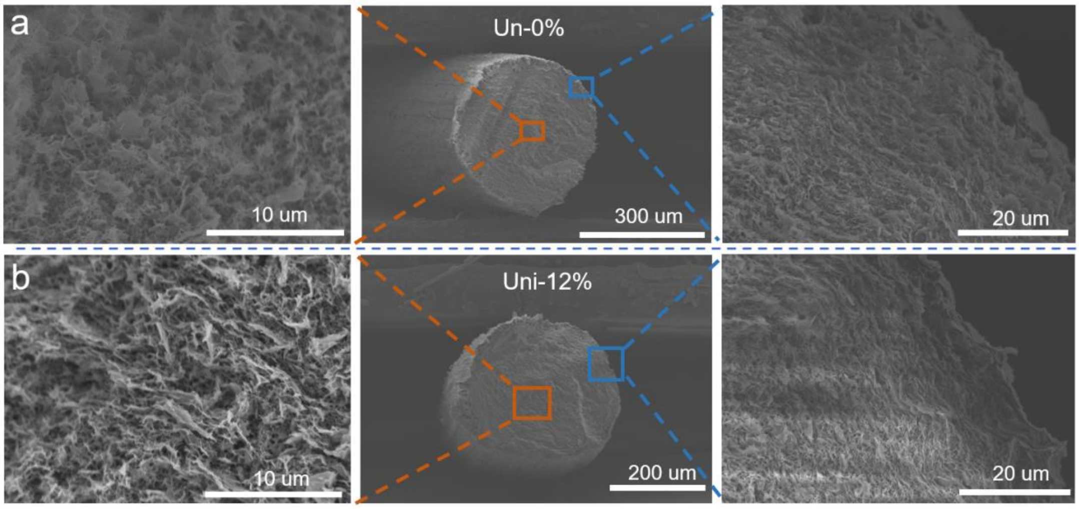

Fig. S7 SEM images showing the cross-sectional morphologies of freeze-dried ANF/BNNSs uniaxial wet filaments (**a**) without and (**b**) with a 12% tensile strain. The uniaxial fibers with a 12% tensile strain only present very slight orientation of BNNSs but no clear densification

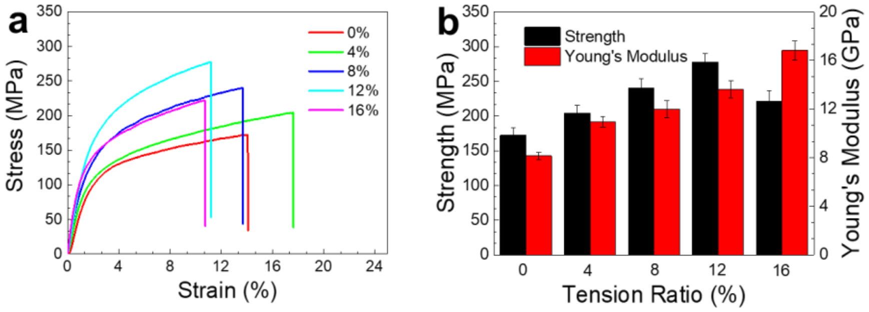

Fig. S8 Tensile stress-strain curve of strained fibers. The tensile strength gets maximum at 12% strain

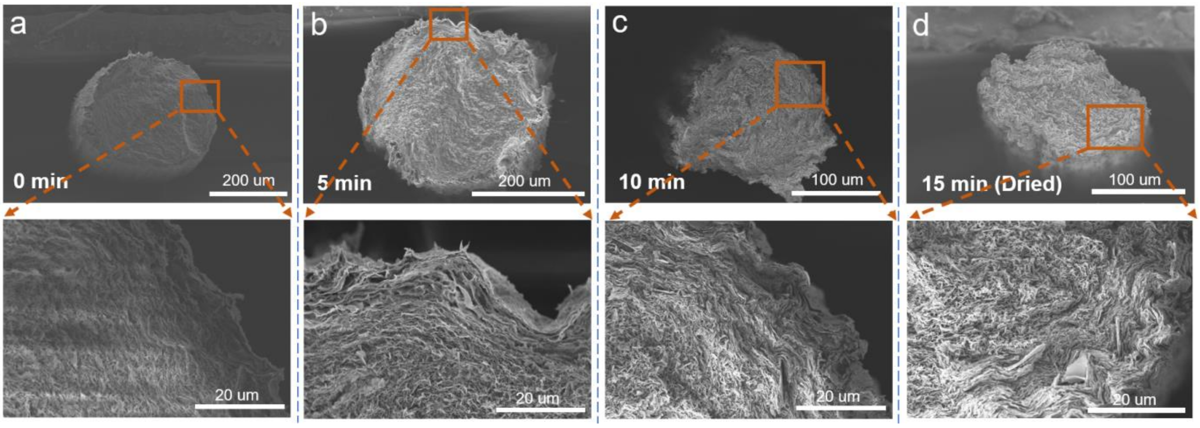

Fig. S9 SEM images showing the cross-sectional morphologies of the freeze-dried uniaxial fibers with a tensile strain of 12% under different dried time. (**a, e**) 0 min; (**b, f**) 5 min; (**c, g**) 10 min; (**d, h**) 15 min

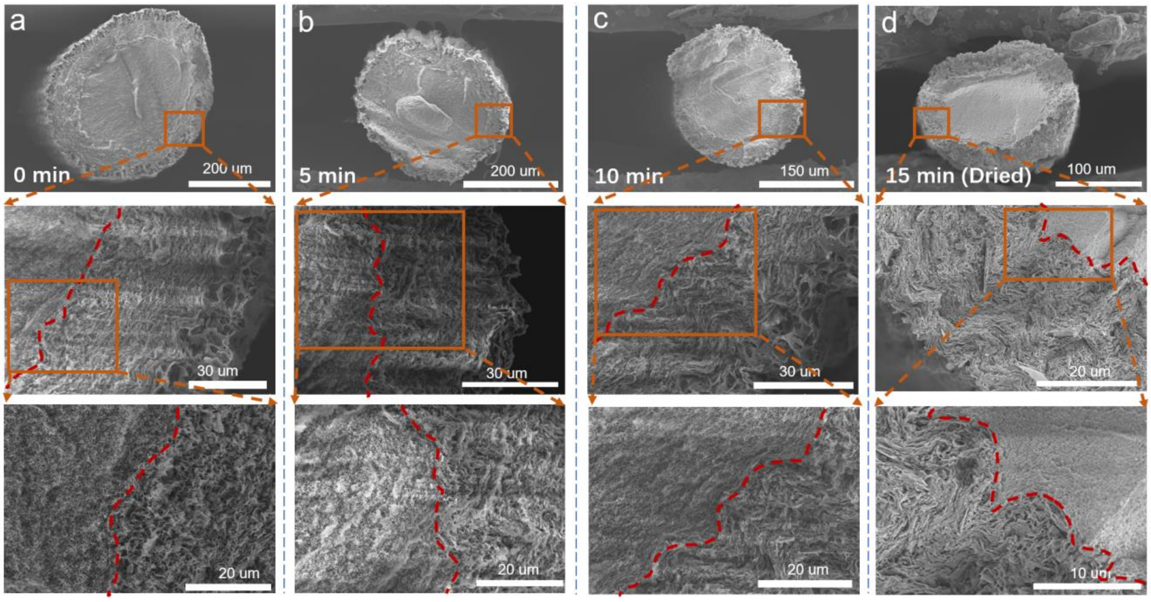

Fig. S10 SEM images showing the cross-sectional morphologies of freeze-dried coaxial fibers with a tensile strain of 12% under different dried time. (**a**) 0 min, (**b**) 5 min, (**c**) 10 min, (**d**) 15min. The red dotted curves show the core-sheath structural interface

a

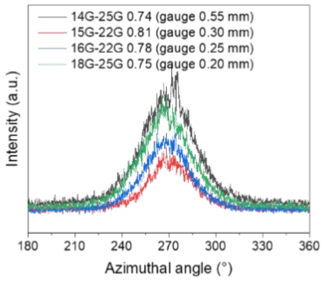

b

	Inner diameter (mm)	Outer diameter (mm)	Wall thickness (mm)
	Outer needle		
14G	1.60	2.10	0.25
15G	1.30	1.80	0.25
16G	1.20	1.60	0.20
17G	1.10	1.50	0.20
18G	0.90	1.30	0.20
	Inner needle		
22G	0.40	0.70	0.15
25G	0.20	0.50	0.15

c

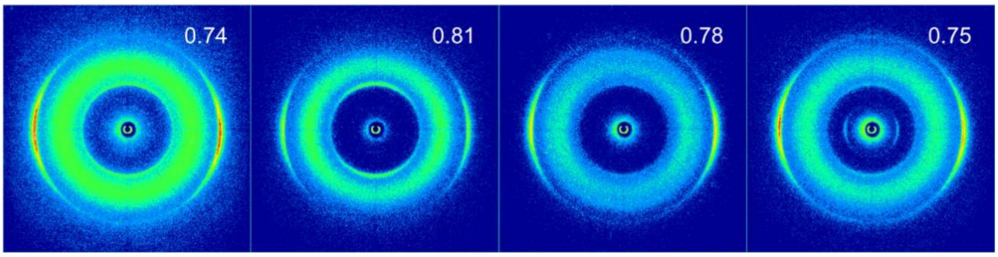

Fig. S11 WAXD tests of different core-sheath content combinations. (**a**) Intensity of azimuthal angle from (**c**) 2D WAXD image at (002) peak. (**b**) Technique parameters of needles used in the experiments. (**c**) 2D WAXD images of core-sheath different combinations (contents)

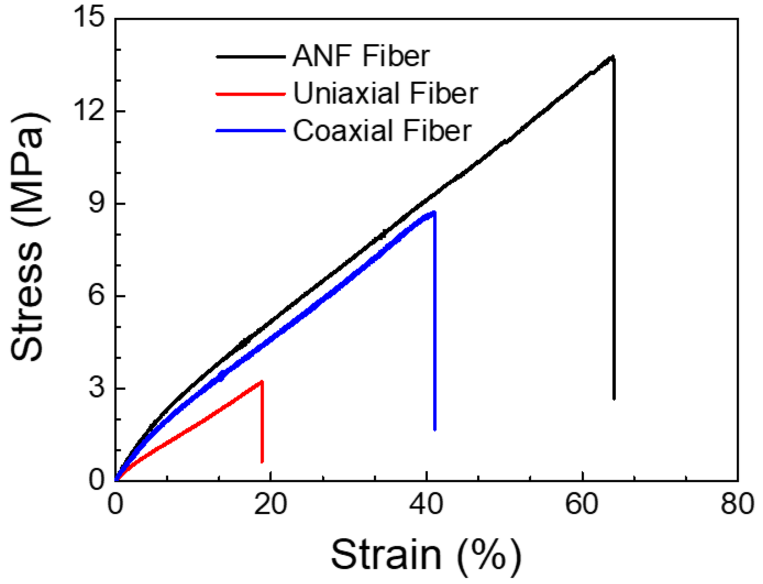

Fig. S12 Typical stress-strain curves of pure ANF, ANF/BNNSs uniaxial and coaxial wet filaments

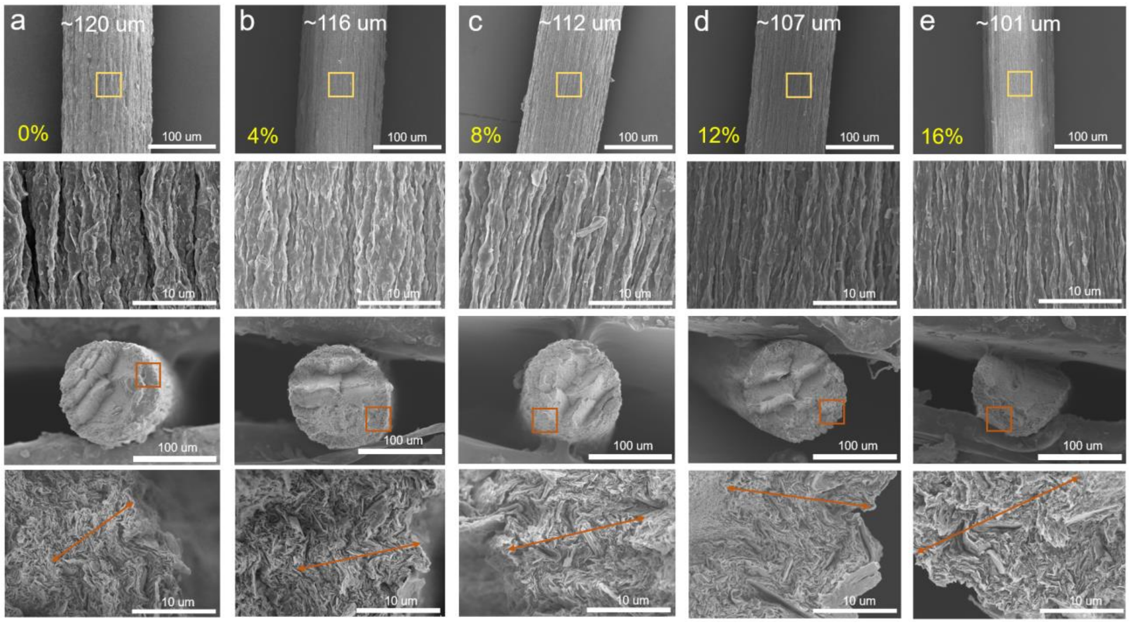

Fig. S13 SEM images showing the BNNSs axial aligning and dense stacking of the coaxial fibers with the increase of tensile strains in the range of 0-16% during hot drawing procedure, (**a**) 0%, (**b**) 4%, (**c**) 8%, (**d**) 12%, (**e**) 16%. The coaxial fibers become slender in diameter with the increase of tensile strain, and more importantly the distinct BNNSs aligning and dense stacking configuration gradually becomes deeper in the sheath until fills the entire sheath when the tensile strain exceeds 8%. This means that the ANF core assisted BNNSs orientation of coaxial fibers should be realized with hot drawing process, and the tensile strain should be large enough. Therefore the 12% tensile strain is adopted in fiber preparation during hot drawing.

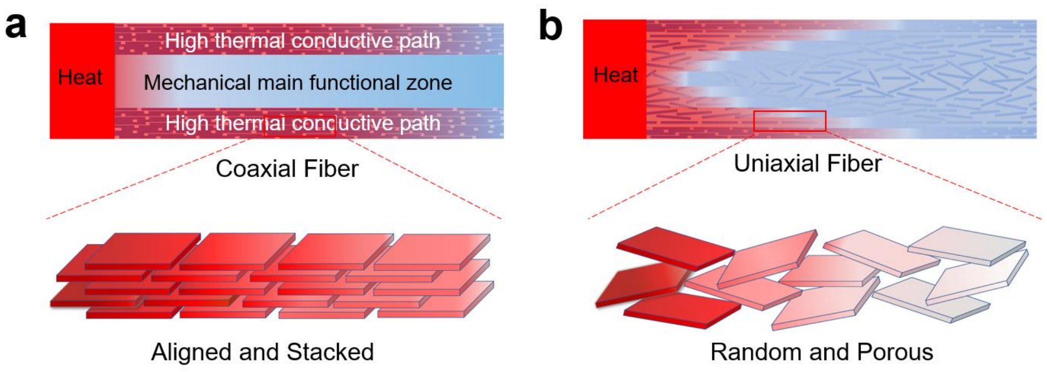

Fig. S14 Schematics illustrating the proposed thermal conduction models of (**a**) the highly axial aligned and stacked BNNSs in coaxial fibers revealing higher thermal conductive performance due to the continuous thermally conductive pathways along the fiber direction, and (**b**) the only surface-depth BNNSs orientation but with random and porous distributed structure of BNNSs in the center of uniaxial BNNSs fibers showing the much weaker thermal conductive performance

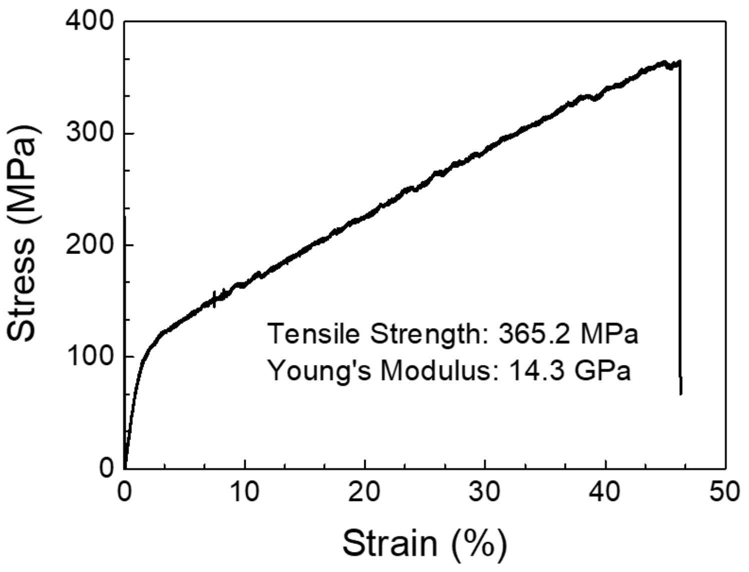

Fig. S15 Stress-strain curve and basic mechanical properties of pure ANF fiber

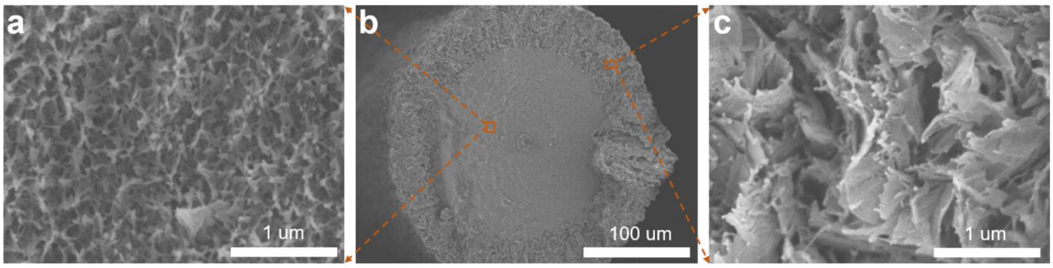

Fig. S16 SEM images showing the cross-sectional morphologies of the tensile fractured ANF/BNNSs coaxial fibers

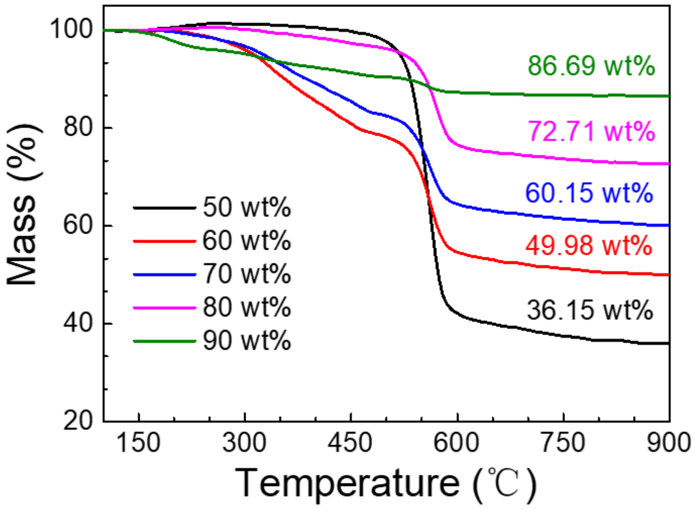

Fig. S17 TGA curves of the coaxial fibers with different outer sheath BNNSs loadings

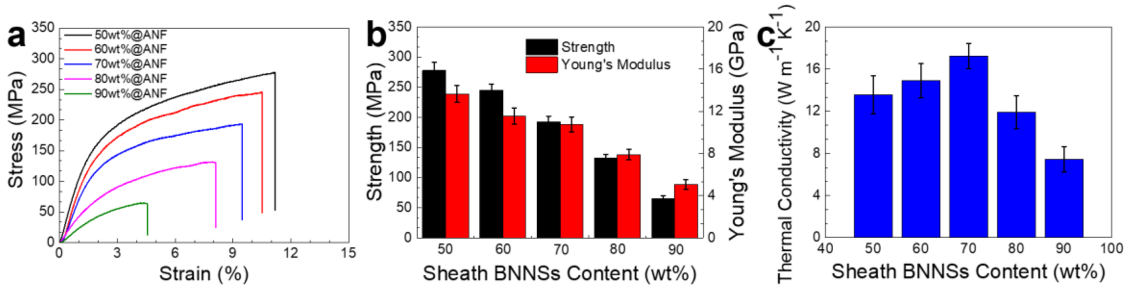

Fig. S18 The mechanical and thermal conductive properties of the ANF/BNNSs coaxial fibers with 50-90 wt% BNNSs sheath loadings. (**a**) Typical stress-strain curves, (**b**) strength and Young's Modulus, and (**c**) thermal conductivities

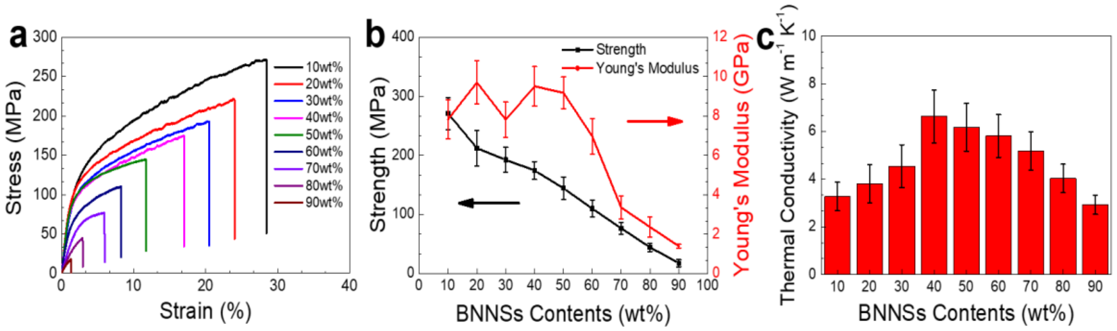

Fig. S19 The mechanical and thermal conductive properties of the ANF/BNNSs uniaxial fibers with 10-90 wt% BNNSs loadings. (**a**) Typical stress-strain curves, (**b**) strength and Young's Modulus, and (**c**) thermal conductivities. The strength and Young's Modulus of the uniaxial fibers gradually decrease with the increase of BNNSs contents and this is mainly attributed to the decreased content of reinforcing ANF. The thermal conductivity of the uniaxial fibers increases first and then decreases with increasing BNNSs loading and the fiber with 40wt% BNNSs fillers reaches the peak value of 6.6 W m^{-1} K^{-1}. When the BNNSs content is lower than 40wt%, the higher

BNNSs loading will lead to more effective construction of BNNSs heat conduction path, so the thermal conductivity of the fibers gradually increases with increasing BNNS loading. While the BNNSs content is over 40wt%, the more porous structure will occur within the fibers (shown in Fig. S18)

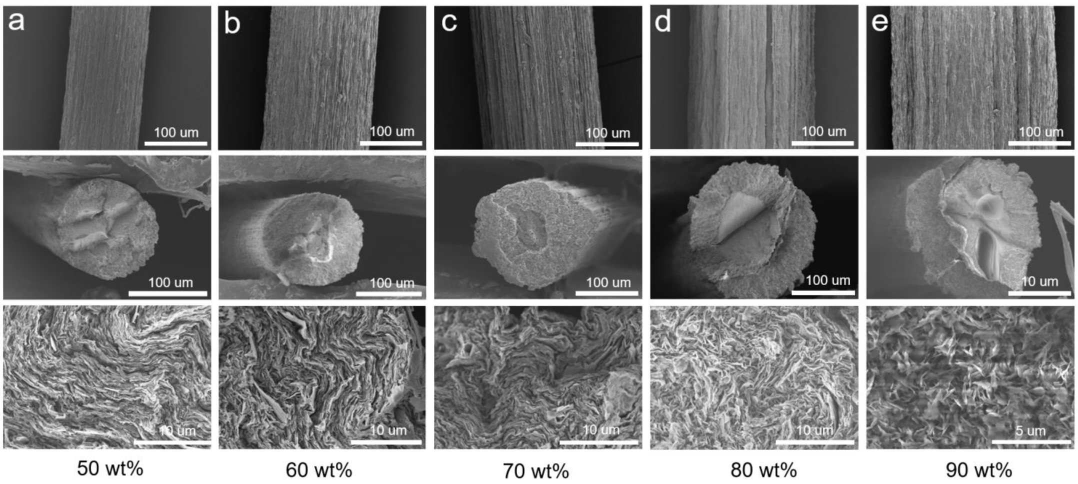

Fig. S20 SEM images showing the cross-sectional morphologies of the ANF/BNNSs coaxial fibers with different BNNSs loading sheaths. **a** 50 wt%, **b** 60 wt%, **c** 70 wt%, **d** 80 wt% and **e** 90 wt%. The fibers become stouter in diameter and rougher in surface with the increase of the outer sheath BNNSs loadings, and the axial aligning and compact stacking patterns of outer sheath BNNSs gradually deteriorate. The stacking patterns of outer sheath BNNSs can still be quite intactly preserved when the BNNSs content reaches 70wt%. As the BNNSs content exceeds 80wt%, the patterns are quickly damaged, especially the random distributed BNNSs and porous space voids can be found

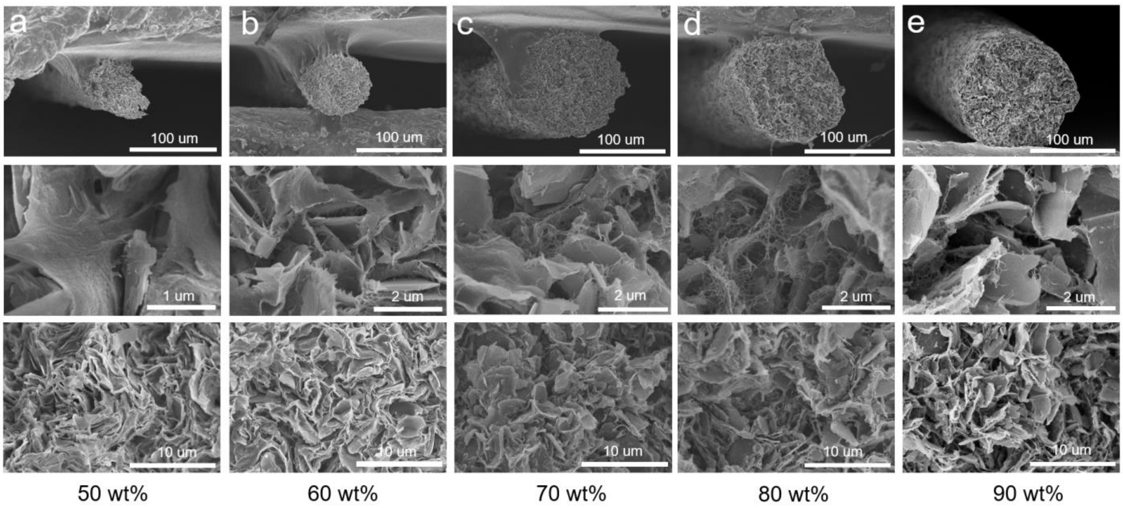

Fig. S21 SEM images showing the cross-sectional morphologies of the ANF/BNNSs uniaxial fibers with different BNNSs loadings. (**a**) 50wt%, (**b**) 60wt%, (**c**) 70wt%, (**d**) 80wt% and (**e**) 90wt%

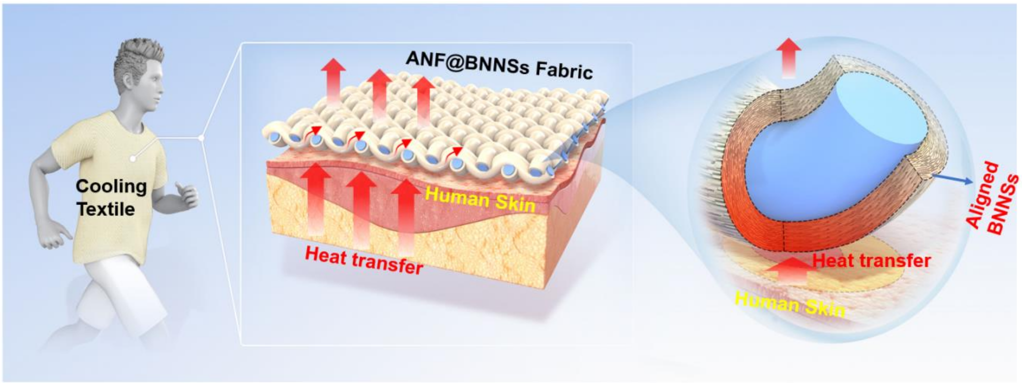

Fig. S22 Schematic thermal regulation illustration of the wearable ANF/BNNSs coaxial fiber textile. The woven textile is made of thermal conductive coaxial fibers with highly axial aligned and dense staked BNNSs in the sheath. The textile can show an extremely effectively heat absorption-conduction-dissipation performance and can continuously release the extra heat produced by the human body along the fiber into the ambient environment [S4]. Note that in real applications, the cooling textile contacts the human body with different angles between the skin and the fibers. This can cause a temperature nonuniformity along the coaxial fabric since the well-aligned and staked BNNSs sheath will induce heat transfer along the fiber-length direction and radial direction

Table S1 Material properties and parameter settings of all components used for finite element modelling

	Inner Core	Outer Sheath	Uniaxial Fiber
Materials	MESH200	SOLID185	SOLID185
Young's Modulus (MPa)	21.53	16.84	16.84
Poisson's Ratio	0.3	0.4	0.4
Density (g cm^{-3})	1.16	1.20	1.20
Diameter (mm)	8	12	12
Length (mm)	30	30	30

Table S2 Compared properties of the as-prepared coaxial BNNSs fibers with other reported works

Composites	BN Content	Young's Modulus (GPa)	Orientation Parameter	Breaking Strength (MPa)	Breaking Elongation	Thermal Conductivity (W/m*K)	References
BN/Cellulose-1	60wt%	4.2	0.27	85	12%	6.3	K. Wu et al.[S1]
	50wt%	4.5	0.21	105	13%	4.2	
	40wt%	5.0	0.15	110	16%	4.1	
	30wt%	4.2	0.13	115	16%	2.3	
	20wt%	5.2	0.11	120	17%	2.0	
	10wt%	6.3	0.06	130	19%	1.8	
BN/TPU-1	60wt%	/	0.519	/	/	5.5	H. Guo et al.[S2]
	50wt%	/	0.461	/	/	4.0	
	40wt%	/	/	/	/	3.0	
	30wt%	/	/	/	/	2.9	
	20wt%	/	/	/	/	1.1	
	10wt%	/	/	/	/	0.7	
BN/Cellulose-2	60wt%	/	0.31	/	/	/	C. Lei et.al[S3]
BN/ANF Film	20wt%	6	/	200	/	/	Rui et al.[S5]
BN/PU-1	5wt%	0.9	/	14	8%	0.262	S. Farajikhah et al.[S6]
	2wt%	0.9	/	11	6%	0.24	
	1wt%	0.9	/	5	5%	0.18	
	0.5wt%	1	/	4	4%	0.15	
BN/PVA	19vol%	10	/	90	0.90%	/	C. S. Boland et al.[S7]
	16vol%	8.3	/	50	0.63%	/	
	14vol%	6.6	/	42	0.65%	/	
BN/TPU-2	30wt%	0.03	/	18	/	1.8	J. Gao et al.[S8]
	20wt%	0.03	/	21	/	1.2	
	10wt%	0.02	/	20	/	0.7	
BN/Cellulose-3	60wt%	/	/	80	13%	3.0	K． Wu et. al[S9]
	40wt%	/	/	105	16%	/	
	20wt%	/	/	120	17%	/	

	10wt%	/	/	132	18%	/	
	5wt%	/	/	130	16%	/	
BN/PI	54wt%	0.5	/	27	45%	5.34	Q. Zhang[S10]
BN/PU-2	40wt%	/	/	/	/	2.6	J. Liu et.al[S11]
	20wt%	/	/	/	/	1.3	
	10wt%	/	/	/	/	0.75	
BN/PDMS	50wt%	/	/	/	/	5.65	Z. Liang et al.[S12]
	21wt%	/	/	/	/	1.5	
	9.3wt%	/	/	/	/	0.7	
	5.3wt%	/	/	/	/	0.18	
BN/ANF Coaxial Fiber	50wt%	13.6	0.81	278	11%	13.5	Our work
	70wt%	10.3	0.67	170	9%	17.2	

Notes: PU, polyurethane; TPU, thermosetting polyurethane; PVA, Polyvinyl alcohol; PI, Polyimide; PDMS, polydimethylsiloxane.

Supplementary References

[S1] K. Wu, Y. Zhang, F. Gong, D. Liu, C. Lei et al., Highly thermo-conductive but electrically insulating filament via a volume-confinement self-assembled strategy for thermoelectric wearables. Chem. Eng. J. **421**, 127764 (2021). https://doi.org/10.1016/j.cej.2020.127764

[S2] H. Guo, H. Niu, H. Zhao, L. Kang, Y. Ren et al., Highly anisotropic thermal conductivity of three-dimensional printed boron nitride-filled thermoplastic polyurethane composites: Effects of size, orientation, viscosity, and voids. ACS Appl. Mater. Interfaces **14**(2), 14568-14578 (2022). https://doi.org/10.1021/acsami.1c23944

[S3] C. Lei, Y. Zhang, D. Liu, X. Xu, K. Wu et al., Highly thermo-conductive yet electrically insulating material with perpendicularly engineered assembly of boron nitride nanosheets. Compos. Sci. Technol. **214**, 108995 (2021). https://doi.org/10.1016/j.compscitech.2021.108995

[S4] T. Gao, Z. Yang, C. Chen, Y. Li, K. Fu et al., Three-dimensional printed thermal regulation textiles. ACS Nano **11**(11), 11513-11520 (2017). https://doi.org/10.1021/acsnano.7b06295

[S5] R. Mo, Z. Liu, W. Guo, X. Wu, Q. Xu et al., Interfacial crosslinking for highly thermally conductive and mechanically strong boron nitride/aramid nanofiber composite film. Compos. Commun. **28**, 100962 (2021). https://doi.org/10.1016/j.coco.2021.100962

[S6] S. Farajikhah, R. Amber, S. Sayyar, S. Shafei, C.D. Fay et al., Processable thermally conductive polyurethane composite fibers. Macromolecular Mater. Engin. **304**(3), 1800542 (2018). https://doi.org/10.1002/mame.201800542

[S7] C. S. Boland, S. Barwich, U. Khan, J. N. Coleman, High stiffness nano-composite fibres from polyvinylalcohol filled with graphene and boron nitride. Carbon **99**, 280-288 (2016). http://dx.doi.org/10.1016/j.carbon.2015.12.023

[S8] J. Gao, M. Hao, Y. Wang, X. Kong, B. Yang et al., 3D printing boron nitride nanosheets filled thermoplastic polyurethane composites with enhanced mechanical and thermal conductive properties. Additive Manufact. **56**, 1-13 (2022). https://doi.org/10.1016/j.addma.2022.102897

[S9] K. Wu, L. Yu, C. Lei, J. Huang, D. Liu et al., Green production of regenerated cellulose/boron nitride nanosheet textiles for static and dynamic personal cooling. ACS Appl. Mater. Interfaces **11**(43), 40685-40693 (2019). https://doi.org/10.1021/acsami.9b15612

[S10] Q. Zhang, T. Xue, J. Tian, Y. Yang, W. Fan et al., Polyimide/boron nitride composite aerogel fiber-based phase-changeable textile for intelligent personal thermoregulation. Compos. Sci. Technol. **226**, 1-8 (2022). https://doi.org/10.1016/j.compscitech.2022.109541

[S11] J. Liu, W. Li, Y. Guo, H. Zhang, Z. Zhang, Improved thermal conductivity of thermoplastic polyurethane via aligned boron nitride platelets assisted by 3D printing. Compos. A: Appl. Sci. Manufact. **120**, 140-146 (2019). https://doi.org/10.1016/j.compositesa.2019.02.026

[S12] Z. Liang, Y. Pei, C. Chen, B. Jiang, Y. Yao et al., General, vertical, three-dimensional printing of two-dimensional materials with multiscale alignment. ACS Nano **13**(11), 12653-12661 (2019). https://doi.org/10.1021/acsnano.9b04202
